# Trait sensitivity to negative feedback in rats is associated with increased expression of serotonin 5-HT_2A_ receptors in the ventral hippocampus

**DOI:** 10.3389/fnmol.2023.1092864

**Published:** 2023-02-09

**Authors:** Paulina Surowka, Karolina Noworyta, Irena Smaga, Malgorzata Filip, Rafal Rygula

**Affiliations:** ^1^Affective Cognitive Neuroscience Laboratory, Department of Pharmacology, Maj Institute of Pharmacology Polish Academy of Sciences, Krakow, Poland; ^2^Department of Drug Addiction Pharmacology, Maj Institute of Pharmacology Polish Academy of Sciences, Krakow, Poland

**Keywords:** feedback sensitivity, animal model, 5HT2A, epigenetic, rat

## Abstract

One of the most important yet still underappreciated mechanisms of depression is distorted cognition, with aberrant sensitivity to negative feedback being one of the best-described examples. As serotonin has been identified as an important modulator of sensitivity to feedback and because the hippocampus has been implicated in the mediation of learning from positive and negative outcomes, the present study aimed to identify differences in the expression of various genes encoding 5-HT receptors in this brain region between the rats displaying trait sensitivity and insensitivity to negative feedback. The results demonstrated that trait sensitivity to negative feedback is associated with increased mRNA expression of the 5-HT2A receptors in the rat ventral hippocampus (vHipp). Further analysis revealed that this increased expression might be modulated epigenetically by miRNAs with a high target score for the *Htr2a* gene (*miR-16-5p* and *miR-15b-5p*). Additionally, although not confirmed at the protein level, trait sensitivity to negative feedback was associated with decreased expression of mRNA encoding the 5-HT7 receptor in the dorsal hippocampus (dHipp). We observed no statistically significant intertrait differences in the expression of the *Htr1a*, *Htr2c*, and *Htr7* genes in the vHipp and no statistically significant intertrait differences in the expression of the *Htr1a*, *Htr2a*, and *Htr2c* genes in the dHipp of the tested animals. These results suggest that resilience to depression manifested by reduced sensitivity to negative feedback may be mediated *via* these receptors.

## 1. Introduction

Depression is one of the most common psychiatric disorders and the leading cause of disability in the 21st century, affecting an estimated 350 million people worldwide (Mulder, 2002; Belzung et al., 2015). One of the most important yet still underappreciated mechanisms of depression is distorted cognition, with aberrant sensitivity to negative feedback (NF) being one of the best-described examples (Beck, 1967, 2008). This phenomenon manifests itself as an overreaction to negative events (catastrophic reaction to perceived failure) and reduced ability to ignore them (Beats et al., 1996; Elliott et al., 1997; Murphy et al., 2003; Taylor Tavares et al., 2008). The inter-individual variability in the sensitivity to NF may represent various types of potential vulnerability to depression and antidepressant treatment (Rygula et al., 2018; Surowka et al., 2022). Thanks to the implementation of the preclinical version of the probabilistic reversal learning (PRL) test, it has become possible to investigate sensitivity to NF in an animal model. Previous research revealed that trait sensitivity to NF is a stable and enduring behavioural trait in rats (Noworyta-Sokolowska et al., 2019), which interacts with antidepressant drugs (Noworyta et al., 2021). As serotonin (5-HT) has been identified as an important modulator of sensitivity to NF (Noworyta et al., 2021) and because the hippocampus (Hipp) has been implicated in the mediation of learning from feedback (Vila-Ballo et al., 2017), the present study aimed to identify differences in the expression of various genes encoding 5-HT receptors in the Hipp between rats displaying trait sensitivity and insensitivity to NF.

To accomplish this, a series of PRL tests were used. We classified each rat as sensitive or insensitive to NF, and subsequently, using reverse transcription-quantitative polymerase chain reaction (RT–qPCR), we analysed intertrait differences in the expression of the *Htr1a*, *Htr2a*, *Htr2c*, and *Htr7* genes in the ventral Hipp (vHipp) and dorsal Hipp (dHipp) of tested animals. After observing significant differences in mRNA levels, we validated these differences at the protein level. Additionally, to identify potential epigenetic mechanisms that could be involved in the observed differences in the expression of 5-HT receptors, we examined relative amounts of microRNAs (miRNAs) for molecules with high target scores for the *Htr2a* gene (*miR-16-5p* and *miR-15b-5p*) in the vHipp, where the altered mRNA and protein levels were observed.

## 2. Materials and methods

### 2.1. Subjects and housing

We used 17 male Sprague–Dawley rats (Charles River, Germany) weighing on arrival 176–200 g, which corresponds to the age of 6–8 weeks and is the standard age in this type of experiment. The sex of animals has been chosen to enable comparison of the results with previous data from ours and other laboratories, which, in the overwhelming majority, have been generated using male rats (for review see (Rygula et al., 2018)). Animals were group-housed (four animals per cage) in an enriched environment (plastic pipes 25 cm long) under controlled temperature (21 ± 1°C) and humidity (40–50%) and a 12 h light/dark cycle (lights on at 7: 00 AM). Throughout the experiment, rats were mildly food restricted to 85% of their free-feeding weight (according to the normal growth curve recommended by the laboratory rodent supplier - Charles River Research Models and Services Catalogue) by providing 15 g of food pellets/rat/day (standard laboratory chow). The food restriction used in our study is a standard and broadly applied procedure in experiments using operant conditioning techniques allowing to maintain motivation and performance of the experimental animals (Rygula et al., 2013; Cieslik et al., 2022; Surowka et al., 2022). Water was always available *ad libitum*. All behavioural procedures were performed during the light phase of the light/dark cycle.

### 2.2. Experimental apparatus

The PRL tests were conducted in operant conditioning chambers (Med Associates; St Albans, Vermont, USA) enclosed within a sound-attenuating box. Each chamber was equipped with a fan, house light, speaker, a food dispenser set to deliver a sucrose pellet (Dustless Precision Pellets, 45 mg; Bio-Serv, New Jersey, USA), and two retractable levers located at the sides of the feeder.

### 2.3. Measuring sensitivity to feedback using the PRL test

After the initial instrumental training described in detail elsewhere (Rygula et al., 2018) and upon reaching the initial training criterion of less than 7.5% omissions on each lever (i.e., less than 15% total omissions but equally distributed between the 2 levers) for 3 consecutive training days, the rats were trained in the PRL paradigm. In brief, each PRL training session lasted until the completion of 200 trials, and each trial lasted for a maximum of 22 s. The start of a trial was signalled by the house light, which remained on until the end of the trial. Two seconds after the trial had started, both levers were presented, and one of them was randomly assigned as the “correct” lever, which delivered a reward (one sucrose pellet) 80% of the times it was pressed. A press on the other lever, the “incorrect” lever, would result in a rewarding outcome only 20% of the times it was pressed. No response in 10 s triggered the intertrial interval (ITI) and was counted as an omission. During the ITI, both levers remained retracted, and the house light was turned off. The same ITI directly followed an unrewarded outcome, i.e., no reward on 20% of the “correct” and 80% of the “incorrect” lever presses. After every 8 consecutive “correct” lever presses (regardless of the outcome), the criterion for the reversal of the outcome probabilities was reached. The previously “correct” lever now became “incorrect” and vice versa. This pattern was followed until the end of the session. The PRL training phase was repeated daily until the individual animals achieved sufficient performance levels. The criteria to be met were a minimum of 3 reversals completed during 3 consecutive training sessions, with less than 15% omissions per session.

### 2.4. Parameters measured in the PRL test

To measure rats’ sensitivity to NF (measured as the ability of animals to ignore infrequent and misleading lack of reward), their decisions were monitored on a trial-by-trial basis. Unrewarded outcomes on the “correct” lever, after which an animal decided to switch levers (probabilistic lose-shifts), were scored and expressed as a ratio of all unrewarded outcomes on that lever. Additional measured parameters included the proportion of all rewarded outcomes followed by a decision to stay with the lever that delivered them (win-stay behaviours), and the number of reversals completed during the test, which relies on the ability to both suppress previously rewarded action and engage in previously unrewarded actions, and was used as a measure of the animal’s performance (Bari et al., 2010).

### 2.5. Feedback sensitivity screening

After achieving a stable performance in the PRL test (a minimum of 3 reversals and less than 15% omissions in three consecutive sessions), animals were subsequently tested in 10 consecutive PRL tests over 10 days. Based on this “NF sensitivity screening,” the rats were divided using the median split into the NF-insensitive and NF-sensitive groups. The division was made based on the average ratio of lever changes following misleading unrewarded outcomes (probabilistic lose-shifts) made by the animals across all 10 screening tests. Because the results of our previous studies have clearly indicated that such a dichotomous categorization based on median split is well suited to investigate NF sensitivity as a stable and enduring cognitive trait in rats (Rygula and Popik, 2016; Noworyta-Sokolowska et al., 2019; Surowka et al., 2020; Noworyta and Rygula, 2021), this method of data analysis has been extended to the present research. The number of screening days following meeting the performance criterion was also based on the results of our previous experiments (Rygula and Popik, 2016; Noworyta-Sokolowska et al., 2019; Surowka et al., 2020; Noworyta and Rygula, 2021).

### 2.6. Brain tissue isolation

After decapitation, the brains were quickly removed, frozen on dry ice and stored at-80°C until processed. The tissue of the dorsal and ventral hippocampi was manually isolated using sterile tweezers by a person experienced in this type of procedure, and according to The Rat Brain Atlas (Paxinos and Watson, 1998). The structures were collected between coordinates from the bregma in mm-dHipp (CA1): AP ~ − 2.6 to −5.2 mm, ML ~ 0 ± 5 mm, DV ~ 3–4.5 mm; vHipp (CA3): AP ~ − 5.2 to −6.04 mm, ML ~4 ± 6 mm, DV ~4.5–9 mm (Paxinos and Watson, 1998).

### 2.7. RNA isolation

The RNA Mini Kit (A&A Biotechnology, Gdańsk, Poland) was used for RNA extraction. The quantity of the RNA was checked with a NanoDrop ND-1000 Spectrophotometer (NanoDrop Technologies Inc., Wilmington, DE, USA).

### 2.8. RT–qPCR for gene expression analysis

The High-Capacity cDNA Reverse Transcription Kit (Thermo Fisher Scientific, Waltham, MA, USA, Life Technologies, Waltham, MA, USA) was used for reverse transcription into cDNA. RT–qPCR was performed by using Quant Studio 3 (Thermo Fisher Scientific, Life Technologies, Waltham, MA, USA) and TaqMan Gene Expression Assays (Applied Biosystems, San Francisco, CA, USA) for *Htr1a* (Rn00561409_s1), *Htr2a* (Rn00568473_m1), *Htr2c* (Rn00562748_m1), and *Htr7* (Rn0056048_m1). The PCR conditions were described previously by Gawlinski et al. (2021). The relative level of mRNA was assessed using the comparative CT method (2^−ΔΔCt^) and normalized to the level of hypoxanthine phosphoribosyltransferase 1 (*Hprt1*), a housekeeping control (Rn01527840_m1).

### 2.9. RT–qPCR for miRNA expression analysis

Total RNA (20 ng) and miRNA-specific stem–loop RT primers (Applied Biosystems, San Francisco, CA, USA) were used for the reverse transcription reactions of miRNA. The cDNAs were then synthesized with the TaqMan MicroRNA Reverse Transcription Kit (Applied Biosystems, San Francisco, CA, USA) according to the manufacturer’s protocol. RT–qPCR was performed with TaqMan MicroRNA assays (Applied Biosystems, San Francisco, CA, USA) to analyse the expression of the following mature miRNAs: *miR-16-5p* (assay ID: 000391) and *miR-15b-5p* (assay ID: 000390). The relative level of miRNA was assessed using the comparative CT method (2^−ΔΔCt^) and normalized to the level of the U6 small nuclear RNA (U6 snRNA). One sample from this analysis was excluded due to technical problems.

### 2.10. Enzyme-linked immunosorbent assay (ELISA)

In the next step, the levels of proteins encoded by the genes that were differentially expressed in rats classified as NF-sensitive and NF-insensitive were measured using ELISA kits (Bioassay Technology Laboratory, Shanghai, China). Quantities of the 5-HT_2A_ (#CAT E1825Ra) and 5-HT_7_ (#CAT E3324Ra) receptors were measured according to the manufacturer’s protocol. Duplicates of each sample and series of standards were transferred to ELISA plates. The absorbance was measured at a wavelength of *λ* = 450 nm using a Multiskan Spectrum spectrophotometer (Thermo LabSystems, Philadelphia, PA, USA). The concentration of proteins was calculated from a standard curve and expressed as ng/mg of protein. For total protein measurement, a bicinchoninic acid assay (BCA) protein assay kit (Serva, Heidelberg, Germany) was used.

### 2.11. Statistical analyses

The data were analysed using GraphPad Prism (version 8.0.1). The distribution of the experimental data was tested using the Kolmogorov–Smirnov test. The screening data were analyzed using one-way (for the whole cohort) or two-way (for the animals classified as sensitive and insensitive to NF) ANOVAs with repeated measures and the within-subject factor of test day (10 levels: test day 1 … test day 10) and between-subject factor of NF sensitivity (2 levels: sensitive and insensitive). For pairwise comparisons, the values were adjusted using the Sidak correction (Howell, 1997). In molecular studies, statistical analyses were performed using a t-Student test. Additionally a Pearson correlation coefficients were computed to assess the linear relationship between the mRNA levels of all investigated genes, and between the levels of miRNAs and the sensitivity to NF (proportion of lose-shift behavior). The tests of significance were performed at *α* = 0.05.

## 3. Results

All animals fulfilled the training criteria and qualified for PRL screening.

### 3.1. NF sensitivity screening

**Figure 1:**
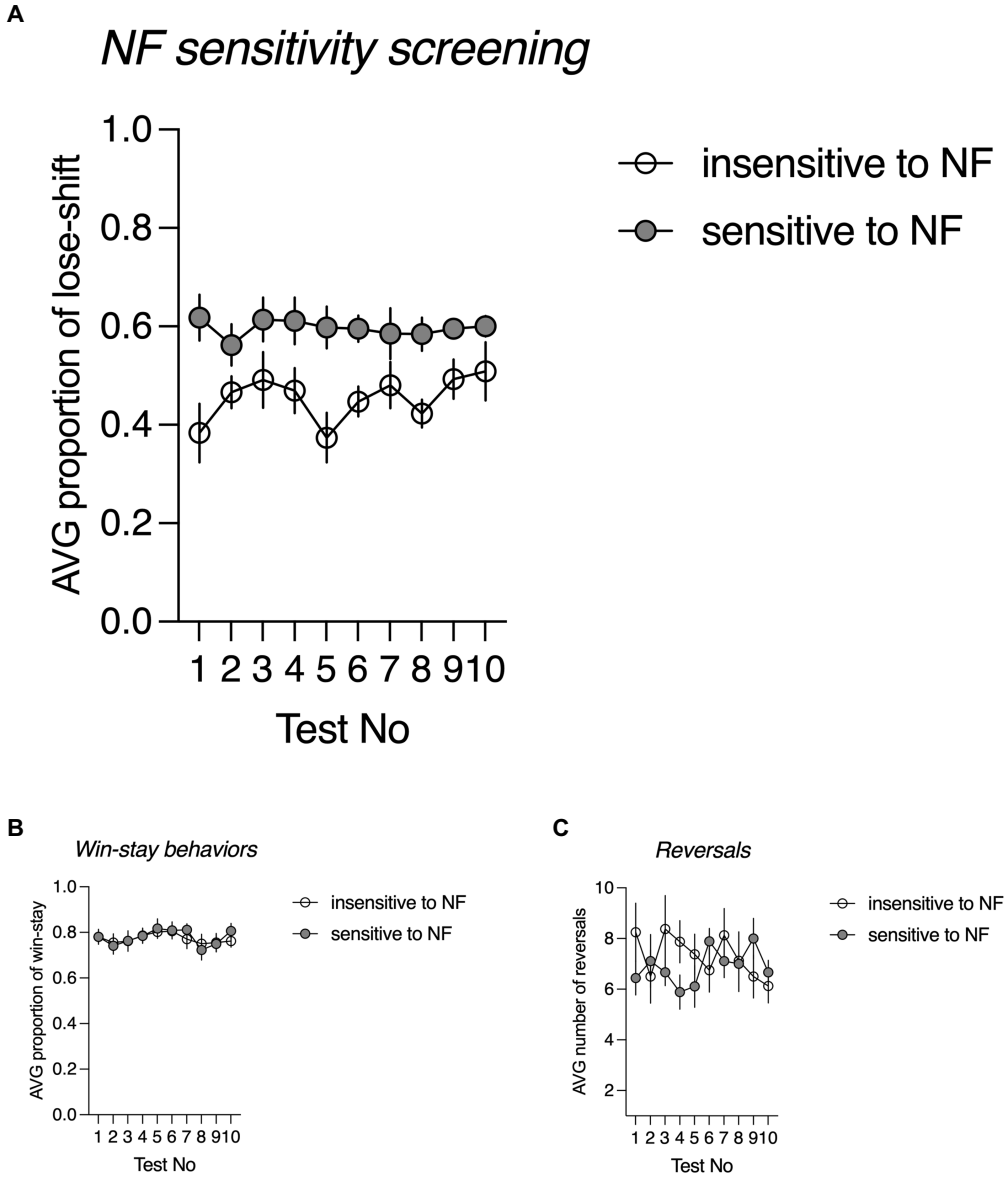
Negative feedback (NF) sensitivity screening. **(A)** The average proportion of lose-shift behaviours following misleading negative feedback in rats classified as trait NF insensitive (open circles, *N* = 8) and trait NF sensitive (filled circles, *N* = 9) across all 10 screening probabilistic reversal learning (PRL) tests; **(B)** The average proportion of win-stay behaviours following positive feedback in rats classified as trait NF insensitive (open circles, *N* = 8) and trait NF sensitive (filled circles, *N* = 9) across all 10 screening PRL tests. **(C)** The average number of reversals in rats classified as trait NF insensitive (open circles, *N* = 8) and trait NF sensitive (filled circles, *N* = 9) across all 10 screening PRL tests.

For the animals classified as NF insensitive, the average proportion of lose-shift responses following misleading NF ranged from 0.385 to 0.505, with an average of 0.454 ± 0.042. For those classified as NF sensitive, the average proportion of probabilistic lose-shift responses ranged from 0.523 to 0.740, with an average of 0.606 ± 0.013. The sensitivity to NF in both subgroups was stable across 10 consecutive screening days {nonsignificant screening Day x NF sensitivity interaction [*F*(9, 135) = 0.853, *p* = 0.569, Figure 1A]}. There were no significant inter-trait differences neither in the proportion of win-stay behaviours [*F*(1, 15) = 0.034, *p* = 0.977, Figure 1B] nor in the number of reversals [*F*(1, 15) = 0.353, *p* = 0.561, Figure 1C].

### 3.2. mRNA levels

**Figure 2:**
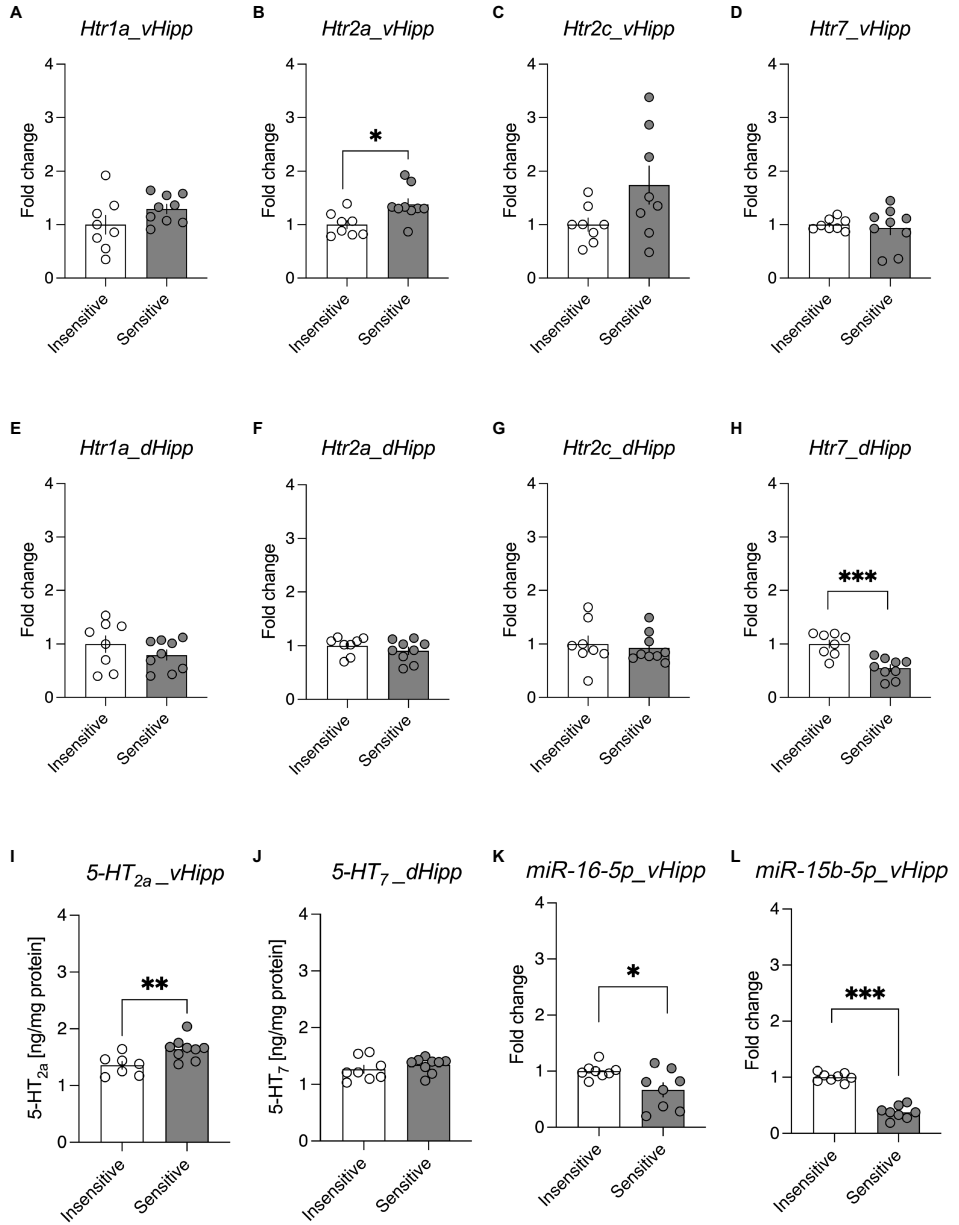
Analysis of the differences in the expression of serotonin 5-HT receptors in the ventral hippocampus (vHipp) and dorsal hippocampus (dHipp) of rats trait insensitive and trait sensitive to negative feedback (NF). Panels **(A–D)** demonstrate the mRNA levels of genes encoding the Htr1a **(A)**, Htr2a **(B)**, Htr2c **(C)**, and Htr7 **(D)** serotonin receptors in the vHipp of trait NF-sensitive and trait NF-insensitive rats. Panels **(E–H)** demonstrate the mRNA levels of genes encoding the Htr1a **(E)**, Htr2a **(F)**, Htr2c **(G)**, and Htr7 **(H)** serotonin receptors in the dHipp of trait NF-sensitive and trait NF-insensitive rats. Panels I and J demonstrate the protein levels of 5-HT_2A_ receptors in the vHipp **(I)** and the protein levels of 5-HT_7_ receptors in the dHipp **(J)** of trait NF-sensitive and trait NF-insensitive rats. Panels K and L demonstrate the levels of miR-16-5p **(K)** and miR-15b-5p **(L)** in the vHipp of trait NF-sensitive and trait NF-insensitive rats. Data are presented as the mean ± SEM. *p < 0.05; **p < 0.01; ***p < 0.001 vs. the NF-sensitive group.

Because each RT–qPCR reaction was performed separately, and because analysis of the correlation between the relative mRNA levels of all analyzed genes revealed no statistically significant correlations between them (see Supplementary Table S1), the intergroup differences between the NF sensitive and NF insensitive animals were analyzed using separate t-tests. In the vHipp of rats classified as NF sensitive, the mRNA level of *Htr2a* was statistically significantly higher than in the vHipp of rats classified as NF insensitive (*t* = 2.886, df = 15, *p* = 0.011, Figure 2B). There was also a positive correlation between the sensitivity to NF (proportion of lose-shift behaviour) and the mRNA levels of *Htr2a* [*r*(15) = 0.670, *p* = 0.003]. There were no statistically significant intergroup differences in the mRNA levels of *Htr1a* (*t* = 1.551, df = 15, *p* = 0.142, Figure 2A), *Htr2c* (*t* = 1.969, df = 14, *p* = 0.069, Figure 2C), and *Htr7* (*t* = 0.438, df = 15, *p* = 0.668, Figure 2D) in this region. The correlation between sensitivity to NF and the mRNA levels of *Htr2c* [*r*(14) = −0.048, *p* = 0.857], and *Htr7* [*r*(15) = −0.048, *p* = 0.857] was not significant. Interestingly, the mRNA levels of *Htr1a*, turned out to be also positively correlated with sensitivity to NF [*r*(15) = 0.499, *p* = 0.041].

In the dHipp, the mRNA levels of *Htr1a* (*t* = 1.155, df = 15, *p* = 0.266, Figure 2E), *Htr2a* (*t* = 1.015, df = 15, *p* = 0.326, Figure 2F), and *Htr2c* (*t* = 0.413, df = 15, *p* = 0.685, Figure 2G) did not significantly differ between the rats classified as NF sensitive and NF insensitive. The level of *Htr7* mRNA in the dHipp was significantly lower in rats classified as NF sensitive compared to their NF insensitive conspecifics (*t* = 4.685, df = 15, *p* < 0.001, Figure 2H). There was also a negative correlation between the sensitivity to NF (proportion of lose-shift behaviour) and the mRNA levels of *Htr7* [*r*(15) = −0.529, *p* = 0.029]. The correlation between sensitivity to NF and the mRNA levels of *Htr1a* [*r*(15) = −0.200, *p* = 0.443], *Htr2a* [*r*(15) = −0.165, *p* = 0.528], and *Ht2c* [*r*(15) = −0.048, *p* = 0.857] was not significant.

### 3.3. Protein levels

After observing significant differences in mRNA levels, these differences were validated at the protein level. Statistically significantly higher levels of the 5-HT_2A_ receptors were detected in the vHipp (*t* = 3.045, df = 14, *p* = 0.009) of rats classified as NF sensitive compared to their insensitive conspecifics (Figure 2I). Surprisingly, no significant intertrait differences in the 5-HT_7_ receptor levels (*t* = 0.941, df = 15, *p* = 0.362) were observed in the dHipp (Figure 2J).

### 3.4. miRNA levels

In the next step, the potential epigenetic mechanism of the observed differences in the expression of mRNA encoding the *Htr2a* gene in vHipp was evaluated by detecting miRNAs (*miR-16-5p* and *miR-15b-5p)*. The miRNA analyses revealed statistically significant lower levels of *miR-16-5p* (*t* = 2.498, df = 14 *p* = 0.0256, Figure 2K) and *miR-15b-5p* (*t* = 12.68, df = 14, *p* < 0.0001, Figure 2L) in the vHipp of rats classified as NF sensitive compared to their NF-insensitive conspecifics. There was also a negative correlation between the sensitivity to NF (proportion of lose-shift behaviour) and the levels of *miR-15b-5p* [*r*(14) = −0.725, *p* = 0.002]. The correlation between sensitivity to NF and the levels of *miR-16-5p* was not significant [*r*(14) = −0.390, *p* = 0.136].

## 4. Discussion

The results of the present study demonstrated that trait sensitivity to NF is associated with increased mRNA expression of 5-HT_2A_ receptors in the rat vHipp. Further analysis revealed that this increased expression might be modulated epigenetically by miRNAs with a high target score for the *Htr2a* gene (*miR-16-5p* and *miR-15b-5p*). Additionally, although not confirmed at the protein level, trait sensitivity to NF was associated with decreased expression of mRNA encoding the 5-HT_7_ receptor in the dHipp. We observed no statistically significant intertrait differences in the expression of the *Htr1a*, *Htr2c*, and *Htr7* genes in the vHipp and no statistically significant intertrait differences in the expression of the *Htr1a*, *Htr2a,* and *Htr2c* genes in the dHipp of the tested animals.

Several previous studies have implicated 5-HT in the modulation of sensitivity to NF. Reports suggest that increasing 5-HT transmission leads to reduced sensitivity to aversive outcomes, whereas decreasing 5-HT transmission, by way of either upregulation of the 5-HT transporter (SERT), presynaptic receptor stimulation, or acute tryptophan depletion, leads to increased sensitivity to NF [reviewed by Rygula et al. (2018)]. In humans, studies by Chamberlain et al. (2006) and Skandali et al. (2018) showed that low, acute doses of the selective 5-HT reuptake inhibitors (SSRIs) citalopram and escitalopram, which were postulated to downregulate 5-HT transmission *via* presynaptic 5-HT autoreceptors, increased the sensitivity to NF in the PRL task. The results from animal models complement these observations. Bari et al. (2010) repeated the effect observed by Chamberlain and collaborators in humans using a preclinical version of the PRL test in rats treated with citalopram. A study by Phillips et al. (2018) revealed that administration of the 5-HT_2C_ receptor agonist (WAY 163909) resulted in a decreased sensitivity to NF in rats. The involvement of 5-HT_2A_ receptors in the mediation of NF has been demonstrated in the past only once by Amodeo et al. (2014). In this study, a 5-HT_2A_ receptor antagonist (M100907) reduced the number of regressive errors in probabilistic reversal learning, which can be interpreted as a reduction in sensitivity to NF. This result is consistent with our observation that low sensitivity to NF is associated with low expression of 5-HT_2A_ receptors in the rat vHipp. Interestingly, it seems that the difference in the expression of 5-HT_2A_ receptors observed between NF-sensitive and NF-insensitive animals is mediated by posttranslational and epigenetic mechanisms associated with differences in the expression of the miRNA with a high target score for the *Htr2a* gene (*miR-16-5p* and *miR-15b-5p*). This absolutely novel result, although pioneering, is not surprising, as it has been shown previously that coping with the stress response is associated with changes in the expression of various miRNAs in the Hipp (Fanselow and Dong, 2010; Floriou-Servou et al., 2018). The reduced expression of *miR-16-5p* and *miR-15b-5p* observed in our study in rats classified as sensitive to NF, which was inversely correlated with the upregulation of *Htr2a* gene expression, was also reported in people suffering from depression, in whom reduced levels of *miR-16* were observed in the cerebrospinal fluid (Artigas, 2013; Song et al., 2015).

Considering that trait sensitivity to feedback can modulate the effects of the SSRI escitalopram (Noworyta and Rygula, 2021) and that 5-HT_2A_ receptor inactivation potentiates the acute antidepressant-like activity of this drug (Quesseveur et al., 2012), the results of our study constitute a matching piece of the puzzle. Namely, the high availability of the 5-HT_2A_ receptors revealed by our study in animals classified as sensitive to NF suggests that vulnerability to depression manifested by increased sensitivity to NF in humans (Elliott et al., 1997; Murphy et al., 2003), and determined by this sensitivity in rats (Surowka et al., 2022) may be mediated *via* these receptors. This finding also suggests the involvement of these receptors in the feedback sensitivity-dependent differences in the effects of acute escitalopram treatment on anxiety-like behaviours described by Noworyta and Rygula (2021).

Given the functional dissociation between the hippocampal regions, where the dHipp performs primarily cognitive functions while the vHipp relates to stress, emotion and affect (Moser and Moser, 1998; Fanselow and Dong, 2010), it is possible that specific changes in the expression of *Htr2a* and *Htr7* in these distinct subregions may serve very different functions. Further, pharmacologically targeted studies, should confirm the exact contribution of the mentioned receptors, along the dorsal–ventral axis of the Hipp, to sensitivity to NF and in depression itself. Moreover, although the 5-HT genes investigated in the present study have been choosen based on the previous reports indicating their involvement in the sensitivity to feedback, depressive disorder or antidepressant therapy (Savitz et al., 2009; Amodeo et al., 2014; Nautiyal and Hen, 2017; Phillips et al., 2018; Raval et al., 2021), one cannot exclude involvement of the other 5-HT receptors (5-HT_1b_, 5-HT_1d_, 5-HT_2B_, 5-HT_3_, 5-HT_4_, 5-HT_6_) in sensitivity to negative feedback. Further studies should address this question, and are needed to determine whether the 5-HT_2A_-dependent effects of trait sensitivity to NF on the efficacy of antidepressant treatment with SSRIs are specific to the Hipp or also occur in other brain regions.

## Data availability statement

The original contributions presented in the study are included in the article/Supplementary material, further inquiries can be directed to the corresponding author.

## Ethics statement

The animal study was reviewed and approved by All experiments were conducted following the European Union guidelines for the care and use of laboratory animals (2010/63/EU). Experimental protocols were reviewed and approved by the 2nd Local Institutional Animal Care and Use Committee at the Maj Institute of Pharmacology Polish Academy of Sciences in Krakow (Permission no. 242/2017).

## Author contributions

All authors listed have made a substantial, direct, and intellectual contribution to the work and approved it for publication.

## Funding

This work was supported by the Polish National Science Centre (Research grants 2018/31/B/NZ7/03690 to RR, and 2016/23/B/NZ4/01562 to RR) and by the statutory funds of the Maj Institute of Pharmacology Polish Academy of Sciences.

## Conflict of interest

The authors declare that the research was conducted in the absence of any commercial or financial relationships that could be construed as a potential conflict of interest.

## Publisher’s note

All claims expressed in this article are solely those of the authors and do not necessarily represent those of their affiliated organizations, or those of the publisher, the editors and the reviewers. Any product that may be evaluated in this article, or claim that may be made by its manufacturer, is not guaranteed or endorsed by the publisher.
