# Supplementary material for: Trait sensitivity to negative feedback in rats is associated with increased expression of serotonin 5-HT2A receptors in the ventral hippocampus
Source: Front Mol Neurosci. 2023 Feb 9;16:1092864. doi: 10.3389/fnmol.2023.1092864 (PMC9948091; doi:10.3389/fnmol.2023.1092864)
Supplement: Supplementary file 1 [file Table_1.DOCX]

Pearson correlation coefficient matrix

vHipp

|  | *Htr1a* | *Htr2a* | *Htr2c* | *Htr7* |
| --- | --- | --- | --- | --- |
| *Htr1a* |  | r=0,074, p=0,777 | r=0,241, p=0,369 | r=-0,170, p=0,514 |
| *Htr2a* | r=0,074, p=0,777 |  | r=0,371, p=0,157 | r=0,457, p=0,0653 |
| *Htr2c* | r=0,241, p=0,369 | r=0,371, p=0,157 |  | r=0,106, p=0,696 |
| *Htr7* | r=-0,170, p=0,514 | r=0,457, p=0,065 | r=0,106, p=0,696 |  |

dHipp

|  | *Htr1a* | *Htr2a* | *Htr2c* | *Htr7* |
| --- | --- | --- | --- | --- |
| *Htr1a* |  | r=-0,016, p=0,952 | r=-0,016, p=0,951 | r=0,321, p=0,209 |
| *Htr2a* | r=-0,016, p=0,952 |  | r=0,178, p=0,494 | r=0,757, p =0,001 |
| *Htr2c* | r=-0,016, p=0,951 | r=0,178, p=0,494 |  | r=0,058, p=0,825 |
| *Htr7* | r=0,321, p=0,209 | r=0,757, p=0,001 | r=0,058, p=0,825 |  |
